# Progressive Medicine—Volume II, 1900

**Published:** 1900-12

**Authors:** 


Progressive Medicine—Volume II, 1900.—A quarterly digest of advances, discoveries and improvements in the medical and surgical sciences. Edited by Hobart Amory Hare, M. D., Professor of Therapeutics and Materia Medica in Jefferson Medical College of Philadelphia. Octovo, handsomely bound in cloth, 401 pages, with 81 engravings. Lea Brothers & Co., Philadelphia and New York. Issued quarterly. Price, $10.00 per year.

The article in this volume on the subject of "Surgery of the Abdomen, Including Hernia," by Dr. Coley, of New York, is statistical and exhaustive, and is by far the best paper that has appeared during the year. The discussion of appendicitis alone is worth several times the volume.

The operation for gastroptosis is discussed, and cuts are used to illustrate cases operated on by Rovsing and Beyea.

The resume by Dr. John G. Clark on "The Treatment of Pelvic Peritonitis," and "The Treatment of Inflammatory Pelvic Exudates," and "The Ultimate Results in Treatment of Retroversions," is especially interesting.

The other departments are of equal value, and cover a large filed. T. J. B.

